# Trait-Based Emotional Intelligence, Body Image Dissatisfaction, and HRQoL in Children

**DOI:** 10.3389/fpsyt.2019.00973

**Published:** 2020-01-23

**Authors:** Olga Pollatos, Eleana Georgiou, Susanne Kobel, Anja Schreiber, Jens Dreyhaupt, Jürgen M. Steinacker

**Affiliations:** ^1^ Clinical & Health Psychology, Institute of Psychology and Education, Ulm University, Ulm, Germany; ^2^ Division Sports and Rehabilitation Medicine, Research Group “Join the Healthy Boat—Primary School”, Ulm University, Ulm, Germany; ^3^ Institute of Epidemiology and Medical Biometry, Ulm University, Ulm, Germany

**Keywords:** emotional intelligence, body image dissatisfaction, body image, health-related quality of life, childhood

## Abstract

**Background:**

Body image dissatisfaction (BID) is related to an increased risk for various health issues including descreased health-related quality of life (HRQoL), the development of problematic eating behaviors and obesity. Previous research indicates that emotional intelligence is one important factor related to BID in adults. Whether this is the case in children, remains yet unknown. Taking this into consideration, the aim of this study was to explore the relationship between BID and trait-based emotion intelligence (TEI) as well as HRQoL in female and male primary school children.

**Materials and methods:**

TEI and BID were assessed via self-reports as well as HRQoL via parental reports in a large sample of 991 primary school children (429 girls) within the “Baden Württemberg Study”, which evaluated the effectiveness of the health prevention programm “Join the Healthy Boat” in Southwestern Germany.

**Results:**

Our findings demonstrated the interrelation between higher levels of TEI and lower levels of BID among girls and boys. Positive associations were found between better HRQoL, better intrapersonal and stress management abilites (subscales of TEI) and lower BID, as reflected by parental and self-reports.

**Conclusions:**

Our results reveal an interconnectivity between TEI, BID, and better HRQoL in female and male primary school children. Although the observed correlations were rather small, they nervertheless support the idea that TEI consists a key-factor for the self-regulation of health-related behavior. Prevention programs could benefit from including processes, that sough to improve aspects of emotional intelligence such as intrapersonal, interpersonal abilities, and adaptability, as an effort of preventing problematic habits or lifestyles that could lead to disordered eating behaviors as well as to obesity in middle childhood.

## Introduction

A thin body ideal is predominant in different cultures, especially in western countries, whilst body image dissatisfaction (BID) in young women is common, causing in some cases disordered eating attitudes and behaviors (1). While the prevalence of these disorders is rising, especially among girls (2), their onset is gradually dropping (3). In this way, school-age children demonstrate discontentment with their body shape (4, 5), something that is often reinforced by the bidirectional dynamic between society and media, in which a thin ideal that rarely fits with reality, is being promoted (6–13).

Body image is a multidimensional construct, which involves perceptions, behaviors, cognitions, and emotions related to individual’s body, that are also connected to the degree of one’s own body image satisfaction and perceptual accuracy (5, 14–16). Furthermore, when trying to understand how body dissatisfaction occurs and evolves through the developmental spectrum, it is worth taking into consideration that body image is strongly related to the holistic experience of embodiment (awareness that my body belongs to me), which reflects the attunement between the inner states (emotions, cognitions etc.) and the body (17–20) and has been found to be present even in early childhood (17, 21). Embodiment can also be regarded as a precondition for social relatedness (22, 23), which plays a central role in children’s developing healthy lifestyle (24). Bearing this in mind, another important characteristic for social relatedness is emotional intelligence [IE; (25)]. More specifically, Mayer and Salovey [(26), pp. 5] define emotional intelligence (EI) as: “the ability to perceive accurately, appraise and express emotion; the ability to access and/or generate feelings when they facilitate thought; the ability to understand emotion and emotional knowledge; and the ability to regulate emotions to promote emotional and intellectual growth.”

In addition, EI not only entails the constellation of personality traits connected to emotions, that include affective personality dispositions, which are determinable *via* self-reports [trait EI (TEI)], but also the self-perceived emotional ability, which can be assessed *via* performance tests [ability EI (AEI); (27–30)]. Accordingly, higher levels of EI are associated with more positive attitudes, more successful relationships, greater adaptability, higher orientation towards positive values as well as less difficulties in expressing, evaluating, and regulating emotions (31–33). Aspects of psychological well-being, such as life satisfaction and happiness, have been found to be related to EI (34), as well as higher levels of subjective physical health (35). Cuesta-Zamora and colleagues (36), demonstrated that low TEI predicted body dissatisfaction as well as eating disorders symptoms in preadolescents and adolescents. At the same time, it was demonstrated that deficits in different aspects of emotional intelligence are positively related to binge eating, body-weight, and body-shape concerns; therefore suggesting an indirect interaction between emotion processing and body perception/image (36).

Moreover, health-related quality of life (HRQoL) describes individual’s perceived quality of life in physical, psychological, and social aspects of health (37), as well as is strongly related to individuals’ subjective well-being (38). Farhat and colleagues (39) found out that girls who overestimated their body-weight, reported at the same time poorer HRQoL than those with accurate body-weight estimations; Although there is a great research interest concerning HRQoL in various children populations suffering from somatic diseases, such as leukemia, paraplegia, heart failure etc. (40–42), little do we know regarding HRQoL and its related psychological parameters.

Bearing these in mind, scope of the current study was to examine the relationship between BID and TEI among 991 primary school children, as well as their interaction related to HRQoL. Based on previous research, we hypothesized that TEI would be associated with lower BID and better HRQoL in children. As BID is known to be more prevalent among girls (43, 44), we sought to lay focus of our analyses also on possible gender differences and thus hypothesized more profound levels of BID among girls.

## Materials and Methods

### Participants and Procedure

After legal guardians provided their written informed consent, 1047 children in the age of 9.59 (*SD* = 0.63) years, were recruited from primary schools (third to fourth grade) in the federal state of Baden-Württemberg (south western Germany) and participated in the Baden-Württemberg Study, which evaluated the health promotion program “Join the Healthy Boat—Primary School” which focuses mainly on the prevention and promotion of a healthier lifestyle among primary school children, in order to increase awareness regarding overweight and obesity (45–48). A more detailed study’s protocol and design have been previously described (45). Study’s approval was obtained from the Ministry of Education as well as from the University’s Ethics Committee.

From the main sample, complete children’s data sets derived from 991 participants; 492 girls (49.6%) and 499 boys (50.4%) with a mean age of 9.58 years (*SD* = 0.62; age ranged from 8 to 11 years). Due to missing data 56 children were excluded from our sample. Finally, there were 787 corresponding parental reports.

### Subjective Reports

For the assessment of trait based emotion intelligence, we used the Bar-On EQi-Youth Version. It is consisted of four subscales: *intrapersonal* (e.g. understanding one’s own feelings) and *interpersonal abilities* (e.g. feeling for someone)*, adaptability* (e.g. adjusting one’s behaviors to changing situations), and *stress management* [e.g. resisting an impulse; (49)]. Due to practical reasons including shortening measurement’s duration we used the shortened version of the Bar-On Emotional Quotient Inventory (EQi)-Youth Version [(49); adapted by (50)]. Good psychometric properties were found by Koch and Pollatos (50) for the German adapted shortened version (CFIs = 1.0, RMSEAs < .001, SRMRs < .001. On the other hand, body image perception was assessed using the Body Silhouette Chart by Collins (51) for preadolescent children. Test-retest reliability coefficient ranged as high as .91 (51). Lastly, parents gave information about their children’s HRQoL [Parent’s Questionnaire KINDL^R^; (52)], by providing information regarding the following aspects: physical well-being (e.g., “my child felt sick”), emotional well-being (e.g., “my child felt insecure”), self-esteem (e.g. “my child didn’t feel much like doing anything”), family (e.g. “my child got on well with us parents”), well-being related to friends/peers (e.g. “my child got along with friends”), and school-related well-being (e.g. “my child was afraid of getting bad grades”). Internal consistency ranges from 0.54 to 0.73 for all subscales and 0.82 for the total score (53).

### Anthropometric Measures

Body mass index (BMI) was calculated (kg/m^2^) and converted to BMI percentiles (BMIPCT) based on national reference data for German children (KIGGS).

### Statistical Analyses

All statistical analyses were conducted using the Statistical Package for Social Sciences (SPSS, version 25). A normality test was performed in order to determine if there was a normal distribution using the Kolmogorov−Smirnov Test. Because of the normal data distribution, parametric tests were carried out. A two-sample t-test was run to compare mean and standard deviation of continuous variables. For the investigation of the relationship between emotional intelligence, BMI, and body image satisfaction as well as HRQoL, there were used regression analyses, Pearson correlations, and partial correlations coefficients. No adjustment for multiple testing was made because of the explorative nature of this study. *p* Values less than 0.05 were considered significant.

## Results

### Sample Descriptives and Questionnaire Data

Sample characteristics on all variables of interest as well as mean scores in trait emotional intelligence, body image dissatisfaction (discrepancy between the real self and the ideal figure) and HRQoL (mean scores, transformed to 0–100) are shown in **Table 1**.

**Table 1:** Sample characteristics and independent samples t-test regarding all variables of interest (total sample: N = 991, respectively N = 787 for parental reports of HRQoL).

	Total sample	Boys	Girls	Boys x Girls	
	*M (SD)*			*t*	*Cohen’s d*
Age (years)	9.58 (.62)	9.61 (0.62)	9.57 (0.62)	—	—
BMI (kg/m²)	17.33 (2.57)	17.16 (2.74)	17.10 (2.72)	*p* > 0.05	—
BMIPCT	48.04 (27.58)	—	—	—	—
TEI total	9.48 (1.61)	9.25 (1.70)	9.70 (1.46)	**p < 0.01	**0.28**
TEI intrapersonal	1.63 (0.66)	1.56 (0.68)	1.70 (0.60)	**p < 0.001	**0.21**
TEI interpersonal	2.22 (0.59)	2.08 (0.62)	2.36 (0.51)	***p < 0.001	**0.24**
TEI adaptability	2.02 (0.66)	2.10 (0.67)	1.94 (0.63)	***p < 0.001	**0.24**
TEI stress management	3.60 (0.75)	3.51 (0.78)	3.69 (0.70)	***p < 0.001	**0.24**
Body Image Dissatisfaction	−0.38 (0.82)	−0.31 (0.83)	−0.45 (0.81)	*p* > 0.05	—
HRQoL emotional well-being	79.16 (11.37)	77.03 (13.10)	77.23 (13.10)	*p* > 0.05	—
HRQoL self-worth	47.67 (13.87)	47.42 (14.27)	48.21 (13.56)	*p* > 0.05	—
HRQoL well-being in the family	67.40 (12.20)	67.22 (12.34)	67.41 (12.56)	*p* > 0.05	—
HRQoL well-being related to friends/peers	61.80 (12.80)	61.64 (14.00)	61.70 (11.66)	*p* > 0.05	
HRQoL school-related well-being	57.05 (15.96)	55.28 (17.78)	59.25 (13.70)	***p < 0.001	**0.25**

All values are mean ± SD unless stated otherwise; BMI, Body Mass Index; BMIPCT, BMI percentiles; SD, Standard Deviation; ***p < 0.001; **p < 0.01; *p < 0.05.

#### TEI and BMI in Boys and Girls

**Figure 1:**
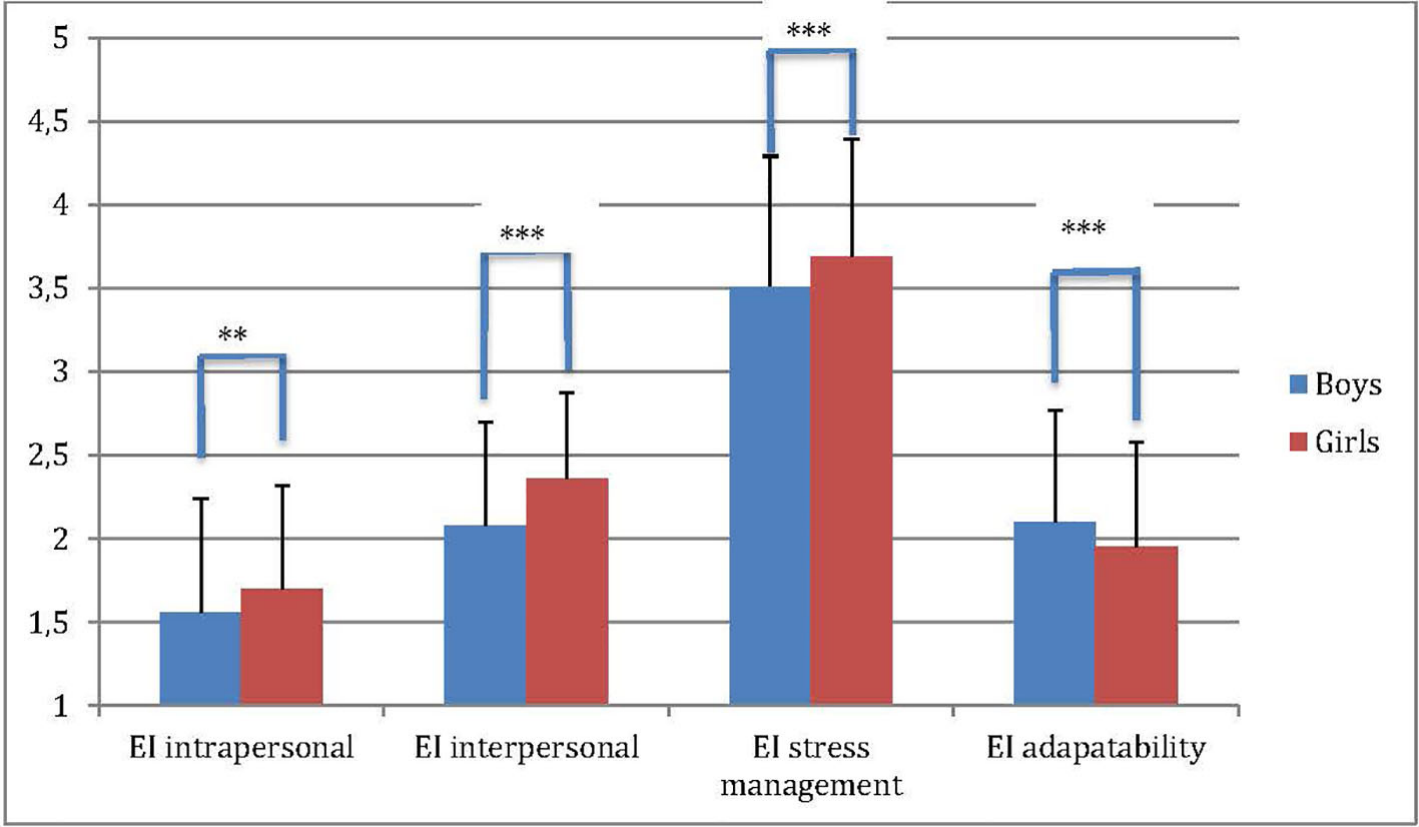
Emotional intelligence scores contrasting boys and girls (**p < 0.01; ***p < 0.001).

*T*-tests for independent samples revealed that girls had significantly higher TEI scores in the subscales intrapersonal [*t*(989) = −3.40, *p* < 0.01], interpersonal [*t*(989) = −7.77, *p* < 0.001] and stress management [*t*(989) = −3.76, *p* < 0.001], while boys scored higher on adaptability [*t*(989) = 3.58, *p* < 0.001] (**Figure 1**).

We observed small, but significant inverse correlations between BMI and trait-based emotional intelligence total score (*r* = −0.12, *p* < 0.001; boys: *r* = −0.15, *p* < 0.001; girls: *r* = −0.09, *p* < 0.05), suggesting that a higher relative body weight was associated with lower emotional intelligence. We therefore controlled for BMI differences in all subsequent analyses.

#### TEI and BID in Boys and Girls

**Figure 2:**
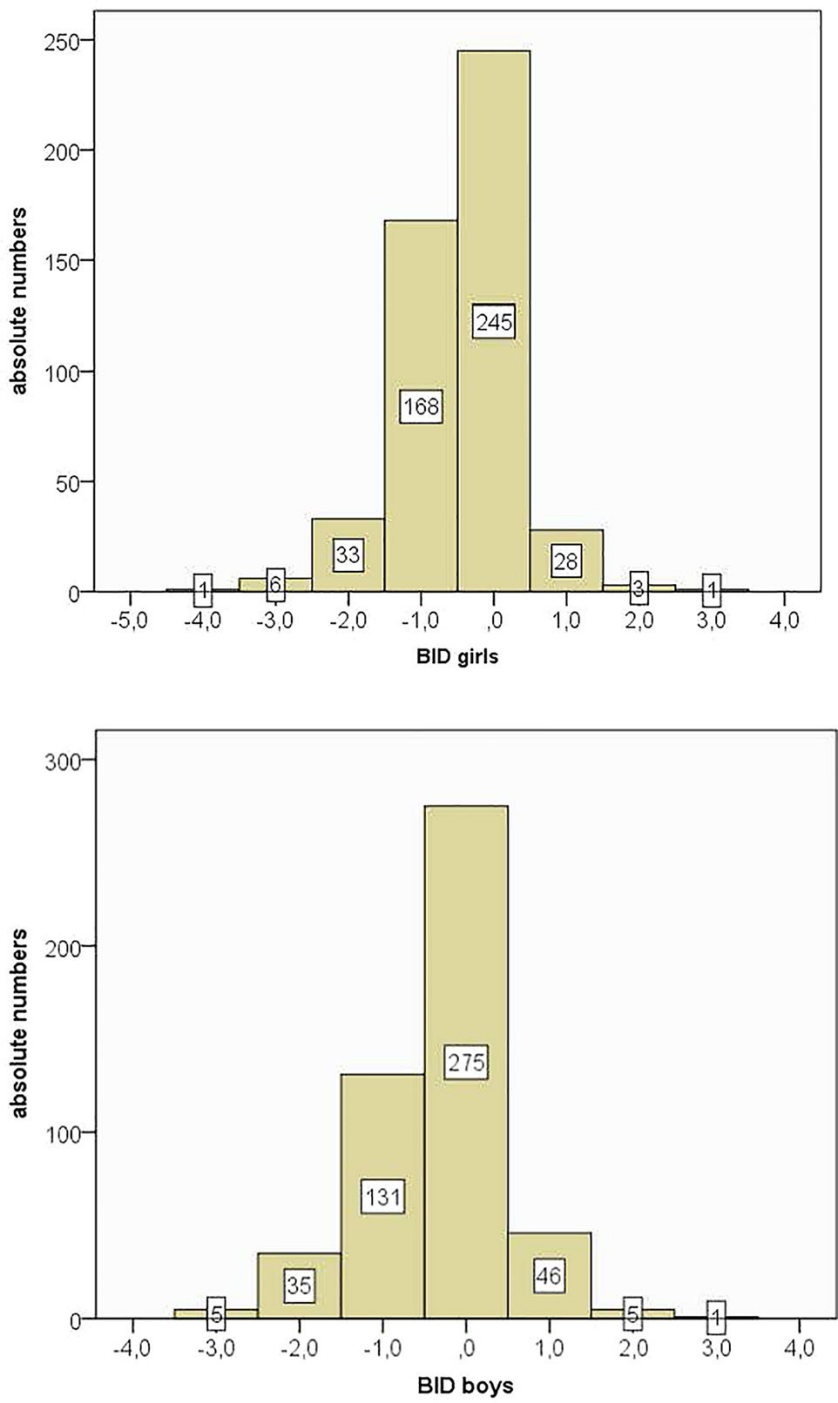
Body image dissatisfaction scores—distribution in boys and girls.

Both girls and boys reported on average a thinner ideal body image than their real self. The distributions of the BID scores demonstrated that 42% of girls and 34% of boys wish to have a thinner body (BID < −0.5). There was a significantly higher BID in girls as compared to boys [see **Figure 2** and **Table 1**; *t* (981) = 2.60, *p* < 0.01].

In a next step we transformed the BID scores into absolute values, with higher scores indicating greater BID. For all further analyses we used absolute scores. We conducted partial correlations between BID and trait-based emotional intelligence (total score) that revealed inverse correlations for both girls (*r* = −0.18, *p* < 0.001) and boys (*r* = −0.15, *p* < 0.01) after controlling for BMI differences. Lower emotional intelligence was therefore associated with higher BID difference scores, thus higher body dissatisfaction.

#### HRQoL, Trait-Based Emotional Intelligence and BID

**Figure 3:**
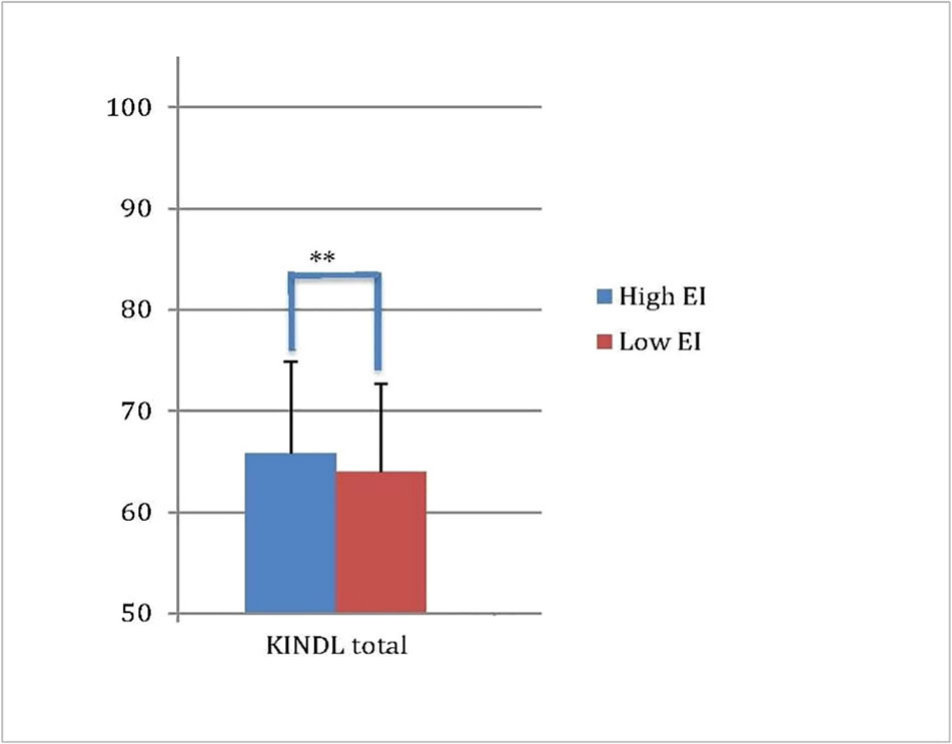
Children's HRQoL (as assessed via the KINDL) in high vs. low emotional intelligence (** *p* < 0.01).

Girls and boys were compared in all aspects of HRQoL except for school-related well-being (girls: mean, 55.36; boys: mean, 58.75; *t*(779) = −2.98, *p* < 0.001). Furthermore, we conducted partial correlations between HRQoL (total score) and emotional intelligence (total score) that revealed a positive relationship (KINDL_total_ _score_ = 0.13, *p* < 0.001) after controlling for BMI differences. Lower emotional intelligence was associated with lower HRQoL (in all three HRQoL measures). To visualize these effects, we compared high and low emotional intelligence groups. As depicted in **Figure 3**, children with higher emotional intelligence had higher HRQoL [KINDL_total_: high EI: 65.8 vs. low EI: 64.0; *t*(777) = −2.77, *p* < 0.01].

We finally carried out a multiple regression analysis (forward selection) to predict HRQoL measures (KINDL_total_ _score_) from the four subscales of trait based emotional intelligence as well as from BID. HRQoL was explained by better stress management (*T* = 3.80, β = .12, *p* < 0.001), better intrapersonal abilites (*T* = 2.70, β = .09, *p* < 0.01, and lower BID (*T* = 2.00, β = 0.06, *p* < 0.05); (*F*(5,985) = 7.81, *p* < 0.001, *R* = 0.19, *R* ^2^ = 0.04).

## Discussion

The present study aimed to elucidate the relationship between trait based emotional intelligence, body image disatisfaction as well as HRQoL in primary school children. In accordance with our hypotheses higher TEI was associated with lower BID in both girls and boys, and these effects remained significant after controlling for BMI. Moreover, HRQoL was predicted by highler levels of stress management, better intrapersonal abilites, as well as by lower BID. However, we did not observe any gender differences concerning HRQoL and its associations to TEI.

These findings are in accordance with prior research, in which it was demonstrated that different dimensions of TEI, but also EI, were closely connected to subjective psychological and physical well-being (27, 34, 35). Likewise, this interaction between EI and body image was indicated in several studies among young athletes, as well as young adults (54–56), but up to now there was no direct evidence among children. Additionally, the observed inverse correlation between TEI and weight status found in this study, is in accordance with prior research showing that obesity is related to disturbed emotion regulation processing among children (57–59).

Taking these into consideration, the close link found between the dimensions of TEI, BID and BMI, ephasizes the need of improving such emotion abilities in schools, as in this way this could have a positive impact on children’s overall HRQoL and well-being. This can be achieved by e.g. integrating, in early interventions, methods in learning how to improve intrapersonal and interpersonal abilities, as well as adaptability; as an effort of preventing both problematic eating attitudes (e.g. obesity) as well as the development of eating disorders in childhood.

There are limitations in our study referring to the fact that our data are the result of a correlational, cross-sectional design, where different causality directions cannot be ruled out. Therefore, longitudinal study designs are necessary to clarify the direction and causal chain of the observed interrelations. Furthermore, the observed correlations were rather small, reflecting the fact that other factors like e.g. emotional regulation abilities associated with TEI might also play a role. As a further shortcoming should be regarded the fact that we used the shortened version of the Bar-On Emotional Quotient Inventory (EQi)-Youth Version adapted by Koch and Pollatos (50) due to practical reasons including minimizing measurement’s duration. Moreover, it has been demonstrated in prior research that the dimensional structure of the EQi- short form is not robust in young people (60). Additionally, the fact that we used parental reports [Parent’s Questionnaire KINDL^R^; (52)] in order to assess HRQoL should be considered as a further shortcoming, which could have led to biased results. Lastly, BID was only assessed *via* the body silhouette chart (51) and not *via* a suitable self-report validated for children in this age group, like for example subscales of the Child Eating Disorder Examination Questionnaire [ChEDE-Q; (61)], that would have been more informative.

In view of these, future longitudinal studies should include further self-report questionnaires with a focus on emotional characteristics associated with both dimensions of emotional intelligence (trait based but also ability based) in children and their interaction to BID and overall well-being. It would be also important to examine in this way further variables, such as emotional intensity or emotional lability as well as other mediation and moderation effects (62). At the same time, data concerning proclivity to mental disorders (such as the SDQ—Strengths and Difficulties Questionnaire) or general cognitive ability should be also taken into account.

To sum up, our study demonstrated for the first time that dimensions of TEI are associated with lower BID and better HRQoL in female and male primary school children. This is the first evidence that shows that already in middle childhood the development of emotional intelligence is related to body image perception. Therefore, more longitudinal studies are needed addressing the developmental pathways of BID not only in childhood but also in adolescence, by taking into consideration emotional intelligence and HRQoL.

## Data Availability Statement

The datasets generated for this study are available on request to the corresponding author.

## Ethics Statement

The Ministry of Education, as well as Ulm University's ethics committee (https://www.uni ulm.de/einrichtungen/ethikkommission-der-universitaet-ulm/) had approved the study. Written informed consent to participate in this study was provided by the participants’ legal guardian/next of kin.

## Author Contributions

OP, SK, AS, EG, and JS participated in the design of the study. SK, AS, JD, and JS carried out the study. OP, EG, and JD performed the statistical analyses. OP and EG drafted the manuscript.

## Funding

The school-based health promotion program “Join the Healthy Boat” and its evaluation study were financed by the Baden-Württemberg Foundation, which had no influence on the content of this paper.

## Conflict of Interest

The authors declare that the research was conducted in the absence of any commercial or financial relationships that could be construed as a potential conflict of interest.

## Abbreviations

TEI, trait based emotional intelligence; HRQoL, health-related quality of life; BID, body image dissatisfaction; BMI, body-mass index; VAS, visual analogue scale.
